# Endovascular management of innominate artery cannulation at the carotid–subclavian bifurcation using a kissing stent technique

**DOI:** 10.1016/j.jvscit.2026.102374

**Published:** 2026-06-20

**Authors:** Sabrina Straus, Sabina M. Sorondo, Keyuree Satam, Kenneth Tran

**Affiliations:** a Division of Vascular and Endovascular Surgery, Department of Surgery, Stanford University School of Medicine, Stanford, CA; b Division of Vascular and Endovascular Surgery, Department of Surgery, UC San Diego (UCSD), San Diego, CA

**Keywords:** Endovascular innominate repair, Kissing stents, Vascular trauma

## Abstract

Iatrogenic innominate artery cannulation during subclavian central venous access is rare but potentially life-threatening, particularly when occurring near the carotid-subclavian bifurcation. A 76-year-old man with polytrauma-sustained inadvertent right subclavian-innominate cannulation. Standard catheter removal carried a high risk of uncontrolled hemorrhage. Endovascular repair was performed using a kissing-stent technique: a Gore Excluder iliac extension limb (W. L. Gore & Associates; 16 × 14.5 mm) was deployed via brachial access, and a 9-mm VIABAHN stent (W. L. Gore & Associates) was used off-label in the carotid artery and reinforced proximally with a balloon-expandable bare-metal stent. Completion angiography confirmed patency and exclusion of the entry site. Kissing-stent deployment, including the novel use of the Gore Excluder limb, safely preserved branch perfusion, ensured durable seal, and avoided sternotomy.

Arterial cannulation during attempted central venous access is a rare but potentially morbid complication, with the subclavian approach carrying increased risk due to the inability to achieve adequate external compression and its proximity to major arterial branches.^1^ Inadvertent cannulation of the innominate artery, is rare, and potentially life-threatening, especially when large-bore catheters are introduced or when arterial entry occurs near the carotid and subclavian bifurcation.^2^ Traditional management has relied on open surgical repair; however, endovascular techniques now offer less invasive alternatives, particularly in critically ill patients and/or those with polytrauma, in whom sternotomy carries significant morbidity.^3^ This report describes an iatrogenic innominate arterial injury during attempted right subclavian vein catheterization successfully repaired using endovascular kissing stents to preserve both carotid and subclavian perfusion, with novel use of a Gore iliac extender limb delivered from the brachial approach in order to obtain adequate sealing. Informed consent was obtained from the patient for the publication of this case report and accompanying images.

## Case description

A 76-year-old man with hypertension, hyperlipidemia, prediabetes, and presented as a trauma transfer after a rollover motor vehicle collision. Initial evaluation at an outside hospital revealed bilateral rib fractures, a T10-T12 ligamentous injury with associated fluid collection, bilateral pneumothoraces requiring chest tube placement, and significant agitation requiring endotracheal intubation. He was subsequently transferred to our facility for higher-level neurosurgical care.

Upon arrival, the patient was admitted to the surgical intensive care unit for management of a 6 mm subdural vs epidural hematoma, thoracic spine injury, and respiratory failure. He remained intubated and sedated and was hemodynamically stable. On hospital day 3, ultrasound-guided right subclavian vein access was attempted using a standard 18-gauge introducer needle, and a 0.035-inch guidewire was advanced. Following placement of a 7F triple-lumen central venous catheter, arterial-appearing blood return was noted, and transduction confirmed arterial waveforms. Given the presence of a large-bore catheter within a noncompressible arterial location, the catheter was left in place to tamponade the arteriotomy and avoid uncontrolled hemorrhage. Computed tomography angiography of the chest confirmed direct cannulation of the lateral aspect of the distal brachiocephalic (innominate) artery, with the catheter tip extending into the aortic arch and a moderate mediastinal hematoma. No pseudoaneurysm or active extravasation was identified (Fig 1, *A*).

**Fig 1:**
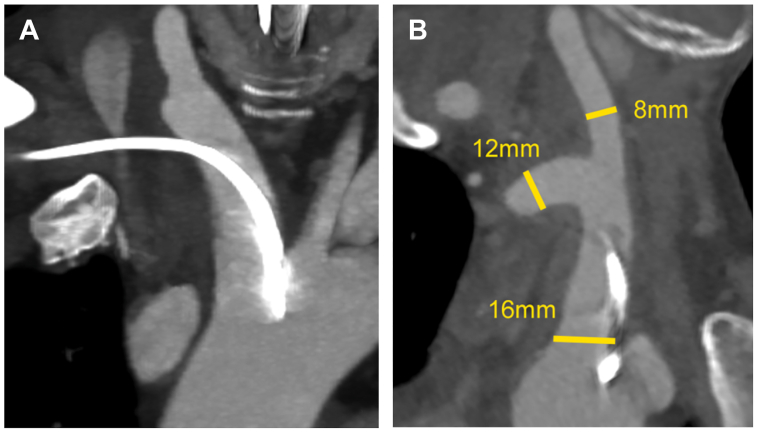
**A,** Inadvertent innominate artery cannulation on lateral aspect of innominate artery near the brachiocephalic confluence. **B,** Diameter measurements of intended endovascular landing zones.

Given that the catheter entry site was located on the lateral aspect of the artery approximately 1 cm proximal to the carotid-subclavian bifurcation, simple catheter removal with manual pressure would be at high risk of uncontrolled hemorrhage into the mediastinum. Achieving an adequate endovascular seal would require covered stents deployed in a “kissing” configuration. The measured diameters of the proximal subclavian, common carotid, and innominate arteries were 12 , 8 , and 16 mm, respectively (Fig 1, *B*). To guide stent sizing, we applied a bifurcation formula based on Finet's law, originally derived from quantitative analysis of coronary artery bifurcations, which relates the diameter of the parent vessel to the sum of its daughter vessels: *D*_innominate artery_ = 0.68 × (*D*_subclavian stent_ + *D*_carotid stent_).^4^ Although this relationship has not been validated for supraaortic vessels, it provides a standardized framework to estimate combined branch diameters in the absence of established formulas for large bifurcations such as the innominate-carotid-subclavian junction. Proportional sizing of the branch stents helps optimize wall apposition and minimize gaps that could predispose to endoleak. Similar applications of fractal-based bifurcation sizing have been described in the common femoral artery, supporting the conceptual utility of this approach beyond coronary anatomy.^5^ In addition to applying this formula, we considered modest oversizing based on the direct vessel measurements and used careful balloon molding to further enhance seal and ensure durable exclusion of the arterial defect. Using the measured innominate diameter of 16 mm, this corresponded to a combined target stent diameter of approximately 24 mm for the subclavian and carotid branches. Based on these calculations, a fully endovascular approach was feasible only with a combination strategy: a Gore iliac extender stent-graft delivered via a brachial approach (eg, 16 mm proximally, 14.5 mm distally) paired with a 9-mm VIABAHN stent-graft.

The patient was brought to the hybrid-operating suite and placed under general anesthesia. Ultrasound-guided right common femoral artery access was obtained, and a 9F shuttle sheath was advanced into the aortic arch. A right brachial artery cutdown was performed, and a Rosen wire was advanced through the subclavian artery into the arch under fluoroscopy. Arch aortography confirmed a right subclavian venous catheter traversing the distal innominate artery near the carotid-subclavian bifurcation. A wire was advanced from the femoral access across the carotid origin, and a 9 × 50-mm VIABAHN stent-graft was positioned in the proximal carotid artery. Concurrently, a 14.5 × 70-mm Gore iliac limb endograft was advanced via the brachial access from the subclavian origin across the innominate artery, spanning the catheter entry site. Both devices were deployed simultaneously in a kissing configuration.

To enhance distal seal, an additional 9 × 50-mm VIABAHN was deployed in the carotid artery. Owing to the differences in radial force between the VIABAHN and the Excluder limb, proximal reinforcement of the carotid stent was performed using a 9 × 39-mm Omnilink balloon-expandable bare-metal stent (Abbott Vascular Inc.), with 3 to 4 mm protruding into the aortic arch to optimize apposition. Final kissing balloon molding was performed using the Omnilink delivery balloon and a Gore molding and occlusion balloon.

After adequate balloon molding and confirmation of stent apposition, the malpositioned central venous catheter was carefully withdrawn. Systemic anticoagulation was not administered given the presence of intracranial hemorrhage. To mitigate thrombotic risk, all sheaths were periodically flushed with heparinized saline, device exchanges were minimized, and wire and catheter manipulation within the aortic arch and supra-aortic vessels was performed with meticulous technique. Completion angiography demonstrated brisk, unobstructed flow through the right common carotid and right subclavian arteries, with no evidence of extravasation (Fig 2). The femoral access site was closed using a Perclose device (Abbott Vascular), and the brachial artery was repaired with the preplaced purse-string suture. The procedure concluded with a palpable radial pulse and no immediate complications. Postoperative computed tomography angiography confirmed widely patent carotid and subclavian stents with complete wall apposition within the innominate artery (Fig 3). Given the patient's intracranial hemorrhage, initiation of dual antiplatelet therapy was deferred. The patient was managed with aspirin 81 mg once deemed safe by the neurosurgical team after repeat computed tomography of the head demonstrated stable intracerebral hemorrhage, with close neurologic and imaging surveillance. At 1-month follow-up, the patient remained on aspirin 81 mg and a statin. Duplex ultrasound study demonstrated continued patency of the carotid and subclavian artery stents without evidence of elevated velocities. The patient remained neurologically intact, with plans for repeat duplex imaging at 6 months.

**Fig 2:**
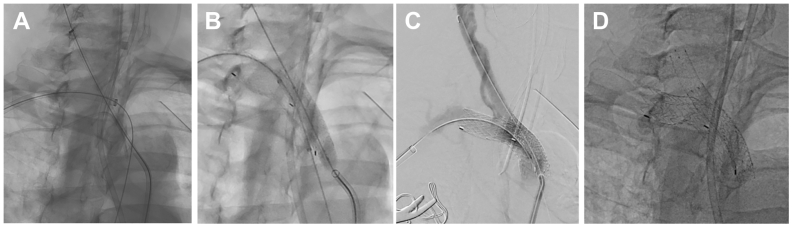
Fluoroscopy-guided repair of innominate arterial injury with kissing stents. **A,** Brachial and femoral access with placement of sheaths across the innominate and carotid arteries. **B,** Postdeployment of both VIABAHN and Excluder limb, with balloon molding around inflated balloon expandable stent. **C,** Angiography demonstrating widely patent stents. **D,** Completion fluoroscopy of stents in final kissing configuration.

**Fig 3:**
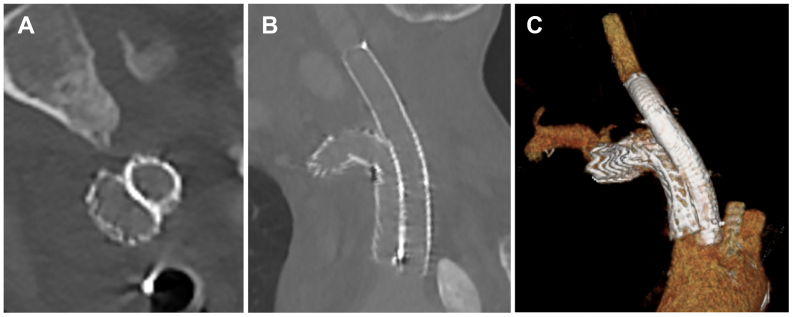
Axial computed tomography images demonstrating D-shaped excluder limb with fully expanded VIABAHN with re-enforcing balloon expandable stent **(A)**; Maximum intensity sagittal projection of kissing innominate-subclavian-carotid stent configuration **(B),** and three-dimensional reconstruction **(C)**.

## Discussion

Management of inadvertent arterial cannulation during central venous access is dictated by several key factors, including catheter size, anatomic location of injury, and patient-specific operative risk. In particular, injuries involving large-bore catheters in noncompressible locations such as the subclavian or innominate arteries require controlled repair, as simple catheter removal may result in catastrophic hemorrhage.^6^, ^7^, ^8^, ^9^ Historically, open surgical repair has been the standard approach for injuries involving the innominate artery, particularly when located near the carotid-subclavian bifurcation where proximal and distal control can be challenging. However, sternotomy carries substantial morbidity in critically ill or polytrauma patients. Endovascular techniques have therefore emerged as a preferred alternative in appropriately selected patients, with previous studies demonstrating lower mortality and complication rates compared with open repair.^10^ Use of percutaneous closure devices such as an Angio-Seal (Terumo Medical Corporation) or Perclose (Abbott Vascular Inc.) has been described, however carries the risk of mal deployment and incomplete hemostasis. Previous reports have described single-limb stent grafting to exclude an arterial injury^11^^,^^12^; however, when the puncture site lies at or near the bifurcation, coverage of a single branch risks cerebral or upper-extremity ischemia. Hybrid approaches have also been reported to address this limitation.^13^ However, this case, an endovascular approach was preferred to avoid as much clamping and heparinization as possible given the patient's subdural hematoma.

Kissing stent reconstruction provides an effective solution for arterial injuries involving the innominate bifurcation, where preservation of both carotid and subclavian perfusion is essential. In this setting, the primary technical challenge is achieving an adequate proximal seal within a large-caliber vessel while maintaining branch patency. Currently available peripheral covered stents are often limited by insufficient diameter and radial force to reliably oppose the innominate artery, particularly when used in a bifurcation configuration. In the present case, this limitation was addressed through a combination strategy that allowed for appropriate diameter matching across the bifurcation. Application of a sizing framework to approximate the required combined branch vessel diameter highlighted the inadequacy of conventional single-device solutions and guided selection of a larger-caliber endograft to achieve seal. The use of an iliac extension limb in this context provided sufficient proximal diameter and conformability to accommodate the innominate artery, whereas simultaneous deployment with a carotid stent-graft enabled preservation of cerebral perfusion. Adjunctive reinforcement of the carotid limb further optimized radial strength and apposition at the bifurcation.

Although endovascular intervention within the supra-aortic circulation, particularly the carotid, carries a risk of cerebral embolization, meticulous wire and catheter manipulation, minimization of device exchanges, and precise stent deployment can effectively mitigate this risk. In this case, completion angiography confirmed preserved carotid patency without flow-limiting complications, and the patient demonstrated an intact neurologic examination following extubation. Dual-access techniques facilitated accurate device positioning, whereas the combination of covered stents and balloon-expandable reinforcement ensured durable seal. This approach highlights an important technical consideration in the endovascular management of supra-aortic injuries: successful exclusion of the defect depends not only on coverage, but on appropriate sizing and force distribution across a complex bifurcation. It is important to note that this technique could be used for bailout stenting if initial attempts at percutaneous closure were not successful. When standard devices are inadequate, adaptation of larger-caliber endografts may provide a viable solution to achieve durable seal while maintaining branch-vessel patency. By enabling safe catheter removal without hemorrhage, preserving cerebral and upper-extremity perfusion, and avoiding sternotomy, this approach demonstrates that endovascular kissing stent repair represents a safe and effective strategy for complex iatrogenic innominate artery injuries.

## Conclusions

Endovascular repair using kissing stents is a safe and effective strategy for managing inadvertent innominate artery cannulation, particularly when the injury is adjacent to the carotid-subclavian bifurcation. In this case, the novel application of a Gore Excluder iliac extension limb enabled appropriate device sizing and durable seal in a large-caliber innominate artery, facilitating successful kissing stent reconstruction. This approach provides reliable exclusion of the arterial entry site while preserving branch vessel perfusion and avoiding the morbidity associated with open surgical repair.

## Funding

None.

## Publication consent

Written informed consent was obtained from the patient for the publication of this case report and accompanying images.

## Disclosures

None.
